# Supplementary material for: lillies: An R package for the estimation of excess Life Years Lost among patients with a given disease or condition
Source: PLoS One. 2020 Mar 6;15(3):e0228073. doi: 10.1371/journal.pone.0228073 (PMC7059906; doi:10.1371/journal.pone.0228073)
Supplement: S1 Appendix — (PDF) [file pone.0228073.s001.pdf]

lillies: an R package for the estimation of excess Life Years Lost among patients with a given disease or condition

O Plana-Ripoll, V Canudas-Romo, N Weye, TM Laursen, JJ McGrath, PK Andersen

S1 Appendix

Basic concepts of survival analysis

Survival and cumulative incidence functions

Survival analysis is used to investigate the distribution of a random variable *T* representing the time from an initiating event to a terminating event, e.g. time from birth to death. Given that it is common to have incomplete observation of *T* because not all study participants are followed until death (i.e. they are censored), survival analysis requires special techniques, such as measuring the survival probability as a function of time *t*:

$$S(t) = \Pr\,(T > t),$$

which can be interpreted as the probability of being alive at time *t*. Supplementary Figure 1a shows a representation of a survival curve *S(t)* from a hypothetical population consisting of all residents in a specific location. By definition, *S(0)=1* because everyone is alive at time 0, and then *S(t)* decreases monotonically as persons start to die, until the last person in this hypothetical population dies at age 105 years, moment in which the survival probability becomes zero (*S(105)=0*).

Analogously, one could calculate the probability of being dead at time *t* (Supplementary Figure 1b), via the cumulative incidence, which is defined as:

$$F(t) = \Pr\,(T \leq t).$$

In addition, we have the relationship $S(t) + F(t) = 1$ for any specific time *t*.

In a competing risks framework, a different number of mutually exclusive causes of death is considered. For simplicity, we will assume that there are two different causes of death. In this situation, the two *cause-specific cumulative incidences* are defined as

$$F_1(t) = \Pr\,(T \leq t, cause = 1) \text{ and } F_2(t) = \Pr\,(T \leq t, cause = 2)$$

which can be interpreted as the probability of dying from cause 1 (or 2) before time *t*. In this situation, the sum of the different probabilities of dying from specific causes ($F_1(t)$ and $F_2(t)$) is equal to the probability of dying from any cause (*F(t)*), and we have therefore the relationship $S(t) + F_1(t) + F_2(t) = 1$ for any specific time *t*. The cause-specific cumulative incidences for the hypothetical population are shown in Supplementary Figure 1c when categorizing deaths into two different groups.

Supplementary Figure 1. (a) Survival curve *S(t)*; (b) cumulative incidence *F(t)*; and (c) cause-specific cumulative incidences $F_1(t)$ and $F_2(t)$ from a hypothetical population consisting of all residents in a specific location.

a) b) c)

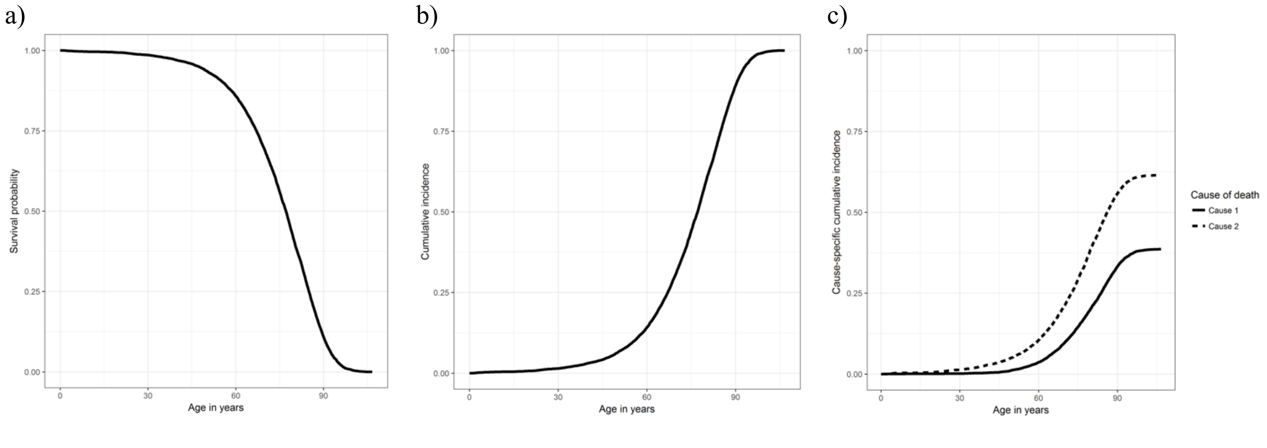

Mean lifetime and number of life years lost

In some situations, it is of interest to summarize the survival function into a single metric, for example the mean survival time (i.e. *mean lifetime*), which can be interpreted as the average life expectancy and is defined as the area under the survival curve:

$$E(T) = \int_0^\infty S(t)dt = e_0.$$

Note that in statistics and epidemiology, the mean lifetime is calculated integrating the survival function $S(t)$ from birth to ∞. However, in demography notation, ∞ is usually replaced by ω, the maximally attainable age; in any case, e_0 will have the same value as long as $S(\omega) = 0$. The mean lifetime for the hypothetical population presented above is $e_0 = 74.5$ years and is shown in Supplementary Figure 2a. However, the mean lifetime is sometimes ill-determined if there are censored observations. Let us assume that this hypothetical population is followed only until age 80 years, moment in which some persons are still alive, and therefore censored. The area under the survival curve observed only until 80 years (Supplementary Figure 2b) cannot be estimated because the curve is not determined after age 80 years. A summary measure of mortality that overcomes this limitation is the *τ-restricted mean lifetime*, which can be interpreted as the average number of years lived before time τ, and is defined by replacing ∞ by τ,

$${}_{\tau}e_0 = \int_0^\tau S(t)dt.$$

Supplementary Figure 2. (a) Survival curve *S(t)* with shaded area under the curve, corresponding to the mean lifetime e_0; (b) survival curve *S(t)* with follow-up truncated at age 80 years; and (c) survival curve *S(t)* with follow-up truncated at age 80 years with shaded area under the curve, corresponding to the 80-restricted mean lifetime ${}_{80}e_0$.

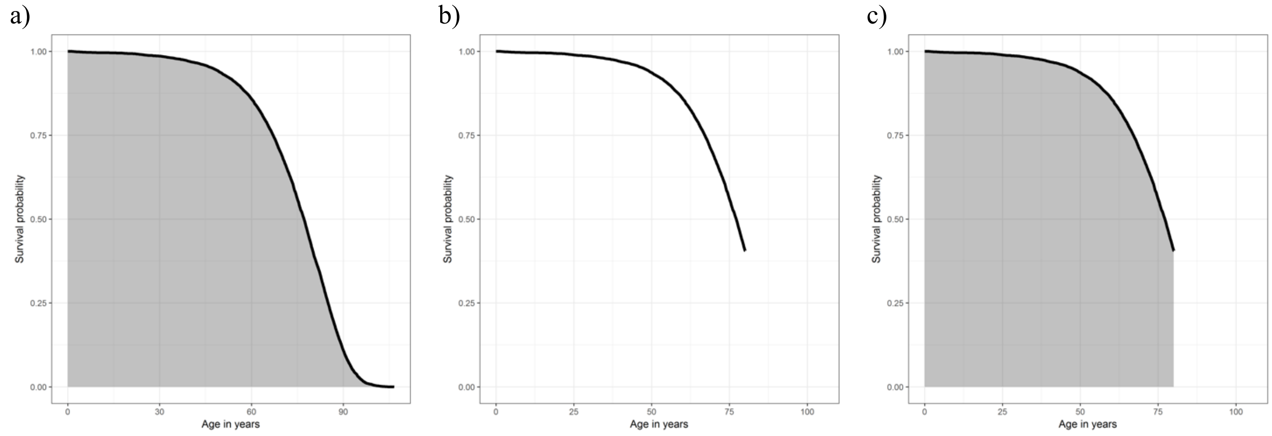

For the hypothetical population presented above, we could estimate the 80-restricted mean lifetime, which is ${}_{80}e_0 = 71.6$ years (Supplementary Figure 2c). It means that persons live on average 71.6 years before age 80 years.

In addition, it follows that the difference between τ and ${}_{\tau}e_0$ can be interpreted as the *expected number of years lost* before time τ,

$${}_{\tau}ə_0 = \tau - {}_{\tau}e_0.$$

For the hypothetical population, persons lose on average 8.4 years before the age of 80 years (${}_{80}ə_0 = 80 - {}_{80}e_0 = 80 - 71.6 = 8.4$).

Given the relationship between the survival and cumulative incidence functions ($S(t) + F(t) = 1$), the expected number of years lost before time τ can also be estimated as the area under the cumulative incidence function (Supplementary Figures 3a and 3b):

$${}_{\tau}\text{ə}_0 = \int_0^{\tau} F(t)dt$$

Supplementary Figure 3. (a) Survival curve *S(t)* with follow-up restricted to age 80 years with shaded area under the curve (${}_{80}e_0$) and above the curve (${}_{\tau}\text{ə}_0$); (b) cumulative incidence *F(t)* with follow-up restricted to age 80 years with shaded area under the curve (${}_{\tau}\text{ə}_0$) and above the curve (${}_{80}e_0$); and (c) cumulative incidences $F_1(t)$ and $F_2(t)$ presented on a 'stacked' plot, that is, $F_1(t)$ and $F_1(t)+F_2(t)$ are plotted against *t*, and ${}_{80}\text{ə}_0^1$ is the area under the lower curve, ${}_{80}\text{ə}_0^2$ the area between the two curves, and ${}_{80}e_0$ is the area above the upper curve.

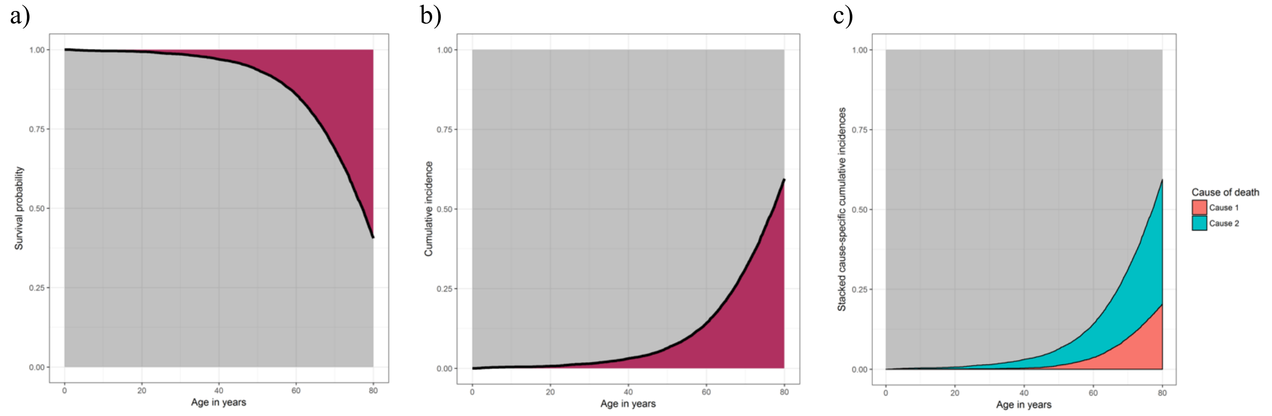

In the competing risks framework, the sum of the different probabilities of dying from specific causes ($F_1(t)$ and $F_2(t)$) is equal to the probability of dying from any cause (*F(t)*), and – following Andersen (2013)[1] – the total number of life years lost ${}_{\tau}\text{ə}_0$ can be decomposed into the number of life years lost due to the 2 mutually exclusive causes of death ${}_{\tau}\text{ə}_0^1$ and ${}_{\tau}\text{ə}_0^2$ as:

$${}_{\tau}\text{ə}_0 = \int_0^{\tau} F(t)dt = \int_0^{\tau}(F_1(t) + F_2(t))dt = \int_0^{\tau} F_1(t)dt + \int_0^{\tau} F_2(t)dt = {}_{\tau}\text{ə}_0^1 + {}_{\tau}\text{ə}_0^2.$$

To sum up, the number of life years lost due to a specific cause of death *j* (possible values *j=1,2*) can be defined as the area under the *j*-cause cumulative incidence:

$${}_{\tau}\text{ə}_0^j = \int_0^{\tau} F_j(t)dt.$$

In the previous example, the total life years lost of 8.4 years can be decomposed into 2.4 years due to cause 1 and 6.0 years due to cause 2. Finally, we have the relationship that the sum of the expected τ-restricted lifetime ${}_{\tau}e_0$, and the number of life years lost before time τ due to cause 1 ${}_{\tau}\text{ə}_0^1$ and cause 2 ${}_{\tau}\text{ə}_0^2$ is equal to τ,

$${}_{\tau}e_0 + {}_{\tau}\text{ə}_0^1 + {}_{\tau}\text{ə}_0^2 = \tau.$$

Graphically, ${}_{\tau}e_0$, ${}_{\tau}\text{ə}_0^1$ and ${}_{\tau}\text{ə}_0^2$ can be represented as follows. The cumulative incidences may be presented on a 'stacked' plot (eFigure 3c); that is, $F_1(t)$ and $F_1(t)+F_2(t)$ are plotted against *t*, and on such a plot, ${}_{\tau}\text{ə}_0^1$ is the area under the lower curve between 0 and τ, and ${}_{\tau}\text{ə}_0^2$ the area between the two curves between 0 and τ. Finally, ${}_{\tau}e_0$ is the area between the upper curve and 1 between 0 and τ. Alternatively, the different curves can be rearranged, as shown in Supplementary Figure 4. The survival curve and the first cause-specific cumulative incidence are presented on a 'stacked' plot, that is *S(t)* and $S(t)+F_1(t)$ are plotted against *t*, and on such a plot, ${}_{\tau}e_0$ is the area under the lower curve between 0 and τ, and ${}_{\tau}\text{ə}_0^1$ the area between the two curves between 0 and τ. Finally, ${}_{\tau}\text{ə}_0^2$ is the area between the upper curve and 1 between 0 and τ.

Supplementary Figure 4. Survival curve *S(t)* with follow-up truncated at age 80 and cumulative incidence for cause 1 $F_1(t)$ presented on a 'stacked' plot, that is, *S(t)* and $S(t)+F_1(t)$ are plotted against *t*. In this graph, ${}_{\tau}e_0$ is the area under the lower curve, ${}_{\tau}\text{ə}_0^1$ is the area between the two curves, and ${}_{\tau}\text{ə}_0^2$ is the area above the upper curve.

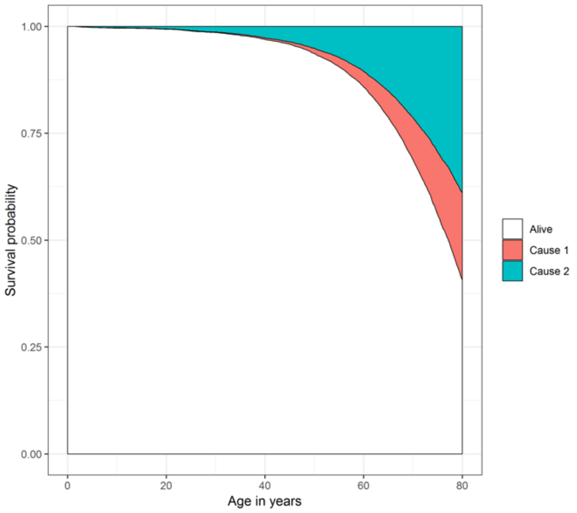

Restricting to persons alive at one specific point in time t_0

In some situations, it is necessary to restrict the analysis to persons alive at one specific point in time. For example, when interested in estimating the survival probability, the mean lifetime, or the number of life years lost for persons with a specific disease that is not diagnosed at birth, it is necessary to restrict the analysis to persons alive at the time of the diagnosis. It is meaningless to estimate the mean life expectancy at birth for persons with a disease, if this diagnosis occurs later in life. Restriction to subjects still alive at time t_0 can be handled simply by considering the conditional survival function $S_{t_0}(t) = \mathrm{S(t)}/S(t_0) = \Pr(T > t \,|\, T > t_0)$ and the conditional cumulative incidence functions $F_{j,t_0}(t) = F_j(\mathrm{t})/S(t_0) = \Pr(T \le t,\ cause = j |\, T > t_0)$, which can be interpreted as the probabilities of being alive (or dead from cause *j*) at time *t* ($t > t_0$) conditioning on being alive at time t_0. Thereby, for those still alive at time t_0, the expected lifetime before time τ is defined by:

$${}_{\tau-t_0}e_{t_0} = \int_{t_0}^{\tau} S_{t_0}(t)dt$$

and the expected number of life years lost before time τ due to cause *j* is defined by:

$${}_{\tau-t_0}\text{ə}_{t_0}^{j} = \int_{t_0}^{\tau} F_{j,t_0}(t)dt$$

For the hypothetical population, 80-restricted lifetime for those alive at age 60 years, ${}_{20}e_{60}$, is 15.6 years, which means they lose 4.4 life years (${}_{20}\text{ə}_{60}$), which can be decomposed into 1.6 years due to cause 1, ${}_{20}\text{ə}_{60}^1$, and 2.8 years due to cause 2, ${}_{20}\text{ə}_{60}^2$. The survival function, cumulative incidence functions, expected lifetime and number of life years lost for the hypothetical population described above when restricting to persons alive at age 60 years is shown in Supplementary Figure 5.

Supplementary Figure 5. (a) Survival curve for those alive at age 60 years $S_{60}(t)$ with follow-up truncated at age 80 years with shaded area under the curve ($_{20}e_{60}$) and above the curve ($_{20}\text{ə}_{60}$); (b) cumulative incidence for those alive at age 60 years $F_{60}(t)$ with follow-up truncated at age 80 years with shaded area under the curve ($_{20}\text{ə}_{60}$) and above the curve ($_{20}e_{60}$); and (c) cumulative incidences for those alive at age 60 years $F_{1,60}(t)$ and $F_{2,60}(t)$ presented on a 'stacked' plot, that is, $F_{1,60}(t)$ and $F_{1,60}(t)+F_{2,60}(t)$ are plotted against t, and $_{20}\text{ə}^{1}_{60}$ is the area under the lower curve, $_{20}\text{ə}^{2}_{60}$ the area between the two curves, and $_{20}e_{60}$ is the area above the upper curve.

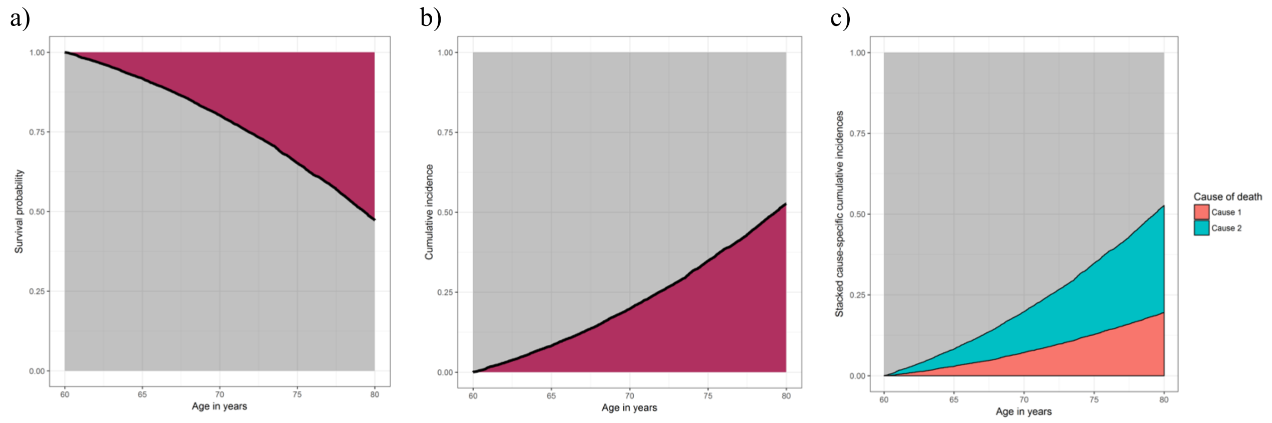

References

1. Andersen PK. Decomposition of number of life years lost according to causes of death. *Stat Med.* 2013;32(30):5278-5285. doi:10.1002/sim.5903
