# Supplementary material for: lillies: An R package for the estimation of excess Life Years Lost among patients with a given disease or condition
Source: PLoS One. 2020 Mar 6;15(3):e0228073. doi: 10.1371/journal.pone.0228073 (PMC7059906; doi:10.1371/journal.pone.0228073)
Supplement: S2 Appendix — (PDF) [file pone.0228073.s002.pdf]

lillies: an R package for the estimation of excess Life Years Lost among patients with a given disease or condition

O Plana-Ripoll, V Canudas-Romo, N Weye, TM Laursen, JJ McGrath, PK Andersen

S2 Appendix

Technical extensions of the Life Years Lost method

S2a: How to assess whether small numbers could influence largely the results 2

S2b: Determine the number of iterations for bootstrap confidence intervals 3

S2c: Interpretation of negative excess Life Years Lost 4

S2a Appendix: How to assess whether small numbers could influence largely the results

The total excess Life Years Lost measure is based on non-parametric survival curves, such as the ones obtained with the Kaplan-Meier estimator. If there is right censoring (as in most of the cases), it could happen that only a small number of persons is at risk of dying after a certain age. For example, assume that 70% of the population is still alive at age 80 years, i.e. $S(80)=0.7$; however, only two persons are still at risk in the specific data available. If one of them dies, the survival curve will immediately drop to 35%. It is important to consider what is an acceptable number of persons at risk n, considering that the weight each of these persons has on the survival curve is $1/n$.

```
LYL <- lyl_range(data = diseased, t0 = age_disease, t = age_death, status = cause_death
, age_begin = 0, age_end = 94, tau = 95)

lyl_checkplot(LYL)
```

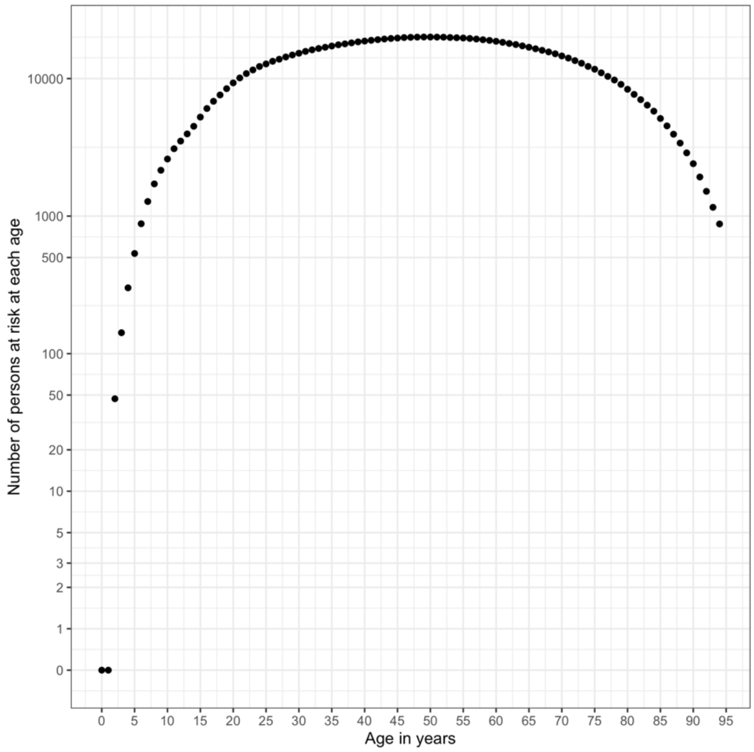

In this example, we see that the number of persons at risk increases rapidly and we have an acceptable number of persons even in the oldest ages of follow-up. Note that small numbers in the early ages are not as problematic as small numbers in the later ages, given that they are not used for many analyses. For example, persons at risk age 1 year, are only included when estimating age-specific Life Years Lost at ages 0 and 1 (and the estimates will have a low weight because they depend on the number of cases). However, persons at risk at age 85 years are included in all age-specific analyses up until 85 years.

S2b Appendix: Determine the number of iterations for bootstrap confidence intervals

We can use non-parametric bootstrap to provide confidence intervals for the estimates.

```
LYL_ci <- lyl_ci(LYL, niter = 1000)

summary(LYL_ci, weights = diseased$age_disease)

## Estimates at ages 0-94 years [maximum age tau = 95 years]
##
## ========================  ========  =======  ========
## \                         estimate  CI_left  CI_right
## ========================  ========  =======  ========
## Remaining life expectancy    34.03    33.89     34.17
## Total Life Years Lost        22.40    22.26     22.53
## - Due to Natural             19.26    19.11     19.39
## - Due to Unnatural            3.14     3.00      3.26
## ========================  ========  =======  ========
## *95% confidence intervals based on 1000 bootstrap iterations
```

In order to assess whether 1,000 simulations are sufficient to ensure a reliable confidence interval, it is possible to draw the estimates considering the first 1, 2, …, 999, or all 1,000 simulations.

```
plot(LYL_ci, weights = diseased$age_disease)
```

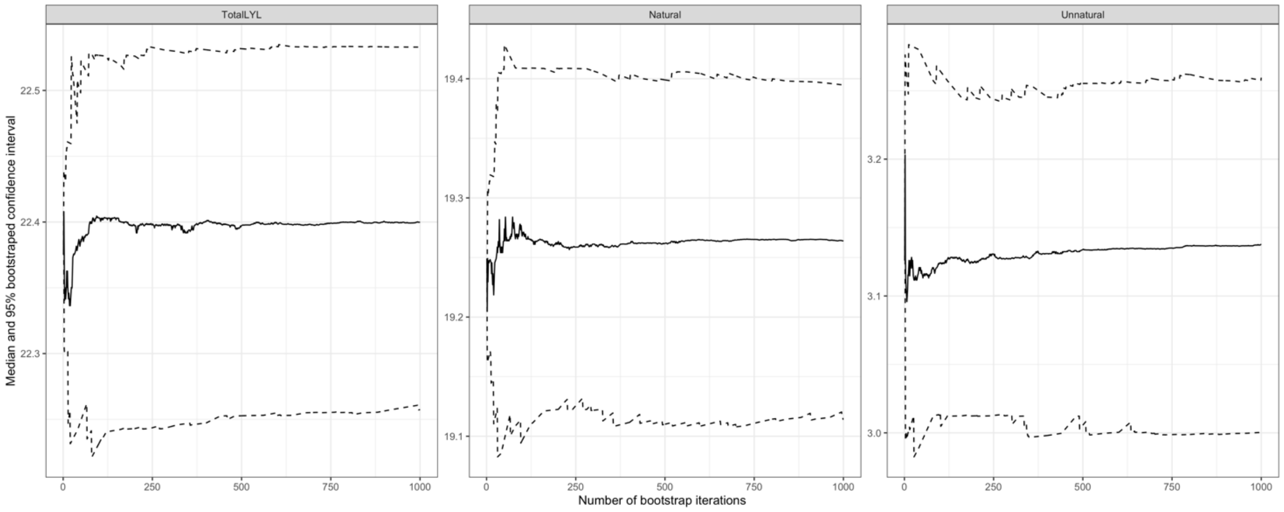

Given that the variability in the estimates is reduced after approximately 400 simulations, it is reasonable to assume that the 95% confidence intervals are accurate. However, a larger number of simulations would provide an even more reliable estimate, especially for Life Years Lost associated with unnatural deaths.

S2c Appendix: Interpretation of negative excess Life Years Lost

The Life Years Lost measure quantifies, for the group of patients with a disease, the average remaining life expectancy from the age at disease onset subtracted from a set reference age, for example 95 years. For the general population, the measure quantifies the average remaining life expectancy subtracted from 95 years for subjects alive at ages corresponding to the age-at-onset distribution for those with the disease. The difference between Life Years Lost of the two groups, the general population and those with the disease, we denote *excess* Life Years Lost, and may be interpreted as the number of years that people with the disorder lose in *excess* of that found in the general population. It could be the case that those with a disease experience less Life Years Lost for some specific causes than those experienced in the general population; for example, it has been shown that those with mental disorders experience less neoplasm-related Life Years Lost than the general population.[1–3] The number of *excess* Life Years Lost due to neoplasms among those with mental disorders is therefore a negative number.

References

1. Erlangsen A, Andersen PK, Toender A, Laursen TM, Nordentoft M, Canudas-Romo V. Cause-specific life-years lost in people with mental disorders: a nationwide, register-based cohort study. *Lancet Psychiatry*. 2017;4(12):937-945. doi:10.1016/s2215-0366(17)30429-7

2. Laursen TM, Plana-Ripoll O, Andersen PK, et al. Cause-specific life years lost among persons diagnosed with schizophrenia: Is it getting better or worse? *Schizophr Res*. 2019;206:284-290. doi:10.1016/j.schres.2018.11.003

3. Plana-Ripoll O, Pedersen CB, Agerbo E, et al. A comprehensive analysis of mortality-related health metrics associated with mental disorders: a nationwide, register-based cohort study. *Lancet*. 2019;394(10211):1827-1835. doi:10.1016/S0140-6736(19)32316-5
